# Reconstruction of the superficial femoral vessels with muscle flap coverage for soft tissue sarcomas of the proximal thigh

**DOI:** 10.1002/micr.30932

**Published:** 2022-06-22

**Authors:** Pauliina Homsy, Ilkka Kantonen, Juho Salo, Anders Albäck, Erkki Tukiainen

**Affiliations:** ^1^ Department of Plastic Surgery University of Helsinki and Helsinki University Hospital Helsinki Finland; ^2^ Department of Vascular Surgery University of Helsinki and Helsinki University Hospital Helsinki Finland

## Abstract

**Background:**

Surgical resection of soft tissue sarcoma with a margin of healthy tissue may necessitate resection and reconstruction of major blood vessels together with soft tissues of the proximal thigh to preserve the limb. The long‐term functional outcomes of these reconstructions remain unestablished. The aim of this report was to assess the vascular and functional outcomes of soft tissue sarcoma patients with femoral vessel reconstructions.

**Patients and Methods:**

Patients who had undergone oncovascular reconstruction during the treatment of proximal thigh soft tissue sarcoma in 2014–2020 were reviewed for details of the vascular and soft tissue reconstructions, and the oncological and functional outcomes. This included eight patients of a median age 59 (range 19–77) years. All had a reconstruction of at least the superficial femoral artery and vein as well as soft tissue reconstruction with a muscle flap. All vessel reconstructions were done with either autologous vein (six grafts/four patients) or allograft (10 grafts/six patients). A microvascular latissimus dorsi flap, with a skin island, was incorporated to cover the vascular grafts in five patients. A pedicled sartorius or gracilis muscle flap was used to fill the defect in three patients.

**Results:**

Graft patency was assessed in seven patients with a median follow‐up of 48 (1–76) months. The arterial graft was patent in 6/8 and the vein graft in 2/8 patients. The gait had returned to normal in five of the six patients assessed. The median MTSS was 70 (43–87)% and the TESS 90 (75–100)%. No local recurrence of the sarcoma was detected.

**Conclusions:**

Vascular reconstruction combined with soft tissue reconstruction enables limb‐sparing surgery in patients with soft tissue sarcoma involving proximal femoral vessels. Although the surgeries are complex with high early morbidity, the achieved long‐term functional outcomes are worthwhile.

## 1 INTRODUCTION

Surgery with tumor‐free tissue margins is essential in achieving local disease control of soft tissue sarcomas (Brennan et al., 2014; Casali et al., 2018; Sampo et al., 2008, 2012). Development of modern surgical techniques, combined with adjuvant treatments has led to limb‐sparing surgery being the standard of treatment for the majority of patients with lower extremity soft tissue sarcoma (Davis et al., 2017; Williard et al., 1992). In cases where the tumor immediately surrounds or invades major blood vessels, the radical excision often still involves sacrifice of the involved vessels. With tumors in the proximal thigh or inguinal region, this has traditionally required proximal amputation, hip disarticulation or hemipelvectomy. Emergence of oncovascular surgery has enabled reconstruction of the resected vessels and salvage of the involved limb (Bonardelli et al., 2000; Davis et al., 2017; Fortner et al., 1977; Fujiki et al., 2020; Ghert et al., 2005; Imparato et al., 1978; Karakousis et al., 1996; Okamoto et al., 2018; Poultsides et al., 2015; Schwarzbach et al., 2005; Song et al., 2009).

The safety and efficacy of vascular reconstruction in conjunction with sarcoma resection has been well established. However, the long‐term graft survival and functional outcomes are sparsely described (Fujiki et al., 2020). In addition, the reported rates of in‐hospital morbidity, wound complications and reoperations are high (Davis et al., 2017; Ghert et al., 2005; Okamoto et al., 2018; Poultsides et al., 2015; Schwarzbach et al., 2005). Incorporation of local or microvascular flaps during the primary operation to improve the soft tissue coverage of the grafts has can be postulated to reduce local complications as well as enable more extensive excisions but the reported cases remain few.

The aim of our report is to assess the early and long‐term outcomes of patients with soft tissue sarcoma in the proximal thigh. In particular, we evaluate the early complications associated with the surgery, the long‐term patency of the vascular reconstructions and the achieved functional outcome.

## 2 PATIENTS AND METHODS

A retrospective analysis of prospectively collected data on patients operated on for deep inguinal or proximal thigh soft tissue sarcoma requiring major vessel reconstruction in conjunction with tumor resection between year 2014 and 2020 was performed. The patient records were reviewed for demographic details, histological diagnosis and grade, details of the surgery and perioperative period, adjuvant treatments, as well as details of any local recurrence or metastases.

**FIGURE 1:**
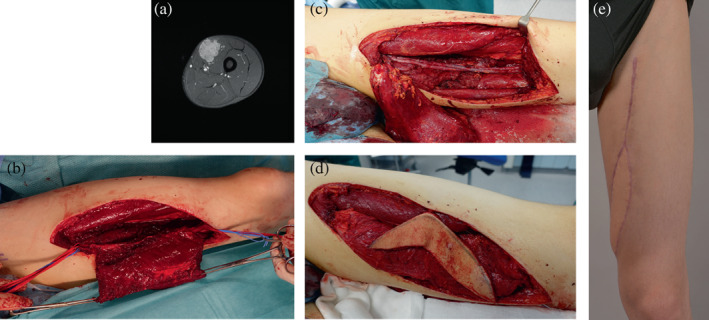
Patient 7. (a) Preoperative T1–weighted axial MRI image of the tumor; (b) perioperative photographs illustrating the resection; (c) reconstructed superficial femoral artery and vein; (d) soft tissue reconstruction with microvascular LD flap to fill the defect cavity; (e) postoperative photograph at 3 months

Eight patients with soft tissue sarcoma were included (Table 1). Six were male. The median age at operation was 59 (19–77) years. Photos of the patient number 7 are shown in Figure 1.

**TABLE 1:** Patient demographics, treatment and follow‐up details of lower limb soft tissue sarcoma patients treated with vascular and soft tissue reconstructions

ID	Age (years) sex	Tumor	Artery resection	Vein resection	Arterial graft material	Vein graft material	Soft tissue reconstruction	Later revision	Graft complications	Resection margins	Adjuvant treatment	Length of follow‐up (months)	Status at the end of follow‐up^c^
Patient 1	77 M	Leiomyosarcoma G3 T2N0M0	EIA ‐ SFA & DFA	EIV ‐ SFV & DFV	Autologous vein	Autologous vein	Pedicled sartorius	Revision and pedicled gracilis due to infection on POD 22		Intralesional	Preoperative radiotherapy	73	Alive with metastases (24 months)
Patient 2	58 F	Leiomyosarcoma G3 T1N0M0	EIA ‐ SFA	EIV – SFV	Allograft	Allograft	Direct closure^a^	Revision due to skin necrosis on POD 21. Prolonged lymphatic leak. LD on POD 64. Tendon transfers for knee extension at 36 months		Marginal	Preoperative radiotherapy^d^	48	Alive with metastases (37 months)
Patient 3	59 M	Leiomyosarcoma G3 T2N0M1	SFA	SFV	Autologous vein	Allograft	Pedicled gracilis	Revision for seroma infection on POD 15	New SFA graft with autologous vein at 19 months	Marginal	Postoperative radiotherapy	43	Alive with metastases
Patient 4	53 F	Leiomyosarcoma G3 recurrence T1N0M0	SFA	SFV	Allograft	Allograft	LD			Marginal	None	36	Death from metastatic disease (13 months). Local recurrence resected at 13 months
Patient 5	29 M	Myoepithelioma GX T3N0M0	SFA	SFV	Allograft	Allograft	Skin graft	LD on POD 105 due to an infected seroma	PTA and stent to proximal arterial stenosis 7 months, 8 times thereafter	Marginal	Postoperative radiotherapy	44	Alive, no evidence of disease
Patient 6	67 M	Myxoid liposarcoma G2 T2N0M0	SFA	SFV	Autologous vein	Allograft	Pedicled Sartorius^a^	Seroma drainage on POD 13	Graft complications	Marginal	Preoperative radiotherapy	30	Alive, no evidence of disease
Patient 7	19 M	Alveolar soft tissue sarcoma GX T2N0M0	SFA	SFV	Autologous vein	Autologous vein	LD	Hematoma evacuation on POD 23		Wide	None	6	Alive, no evidence of disease
Patient 8	75 M	Synovial sarcoma metastases	EIA ‐ SFA	EIV – SFV	Allograft	Allograft	LD^b^	LD harvest site hematoma evacuation on POD 34. Ureter compression requiring pyelostoma on POD 41	Vein graft thrombectomies on POD 2 and 13. Thrombus on POD 33, treated conservatively	Marginal	Postoperative radiotherapy	2	Alive, no evidence of disease

*Note*: Grades presented according to the who classification of tumors of soft tissue and bone (Cancer IAfRo, 2013).

Abbreviations: DFA, deep femoral artery; DFV, deep femoral vein; EIA, external iliac artery; EIV, external iliac vein; F, female; LD, latissimus dorsi microvascular musculocutaneous flap; M, male; POD, postoperative day; PTA, percutaneous transluminal angioplasty; SFA, superficial femoral artery; SFV, superficial femoral vein.

^a^ Synthetic mesh.

^b^ Composite mesh used for inguinal ligament reconstruction and abdominal wall support.

^c^ Time of metastases detection reported in brackets.

^d^ Postoperative chemotherapy was not given due to delayed wound healing.

Six patients were treated for a primary tumor; three leiomyosarcomas, one myxoid liposarcoma, one myoepithelioma and one alveolar soft tissue sarcoma. The patient number 4 had a recurrent leiomyosarcoma treated 3 years earlier with marginal excision and postoperative radiotherapy. The patient number 8 was now operated on for synovial sarcoma metastases after having had the primary tumor treated with wide excision already 35 years ago. His primary tumor had been at the distal thigh and reconstruction had involved a free innervated latissimus dorsi (LD) flap and a common peroneal nerve reconstruction with a sural nerve graft. No local recurrence had been detected but inguinal lymph node metastases have been resected 29 years ago and again 8 months prior to this presentation. The patient number 3 had small, stable lung metastases at time of surgery while the other patients had local disease.

### 2.1 Surgical technique

A radical resection extending to a natural tissue barrier or at least 5 cm away from the tumor was done, including the affected vessels. The superficial femoral artery and vein were reconstructed in all patients. The reconstruction included the external iliac vessels in three patients, one of whom had also the deep femoral vessels reconstructed (Table 1). Smaller branches of the deep femoral artery were reconstructed when assessed beneficial. All reconstructions were done with either autologous vein (six grafts in four patients) or allograft (10 grafts in six patients). The preferred autograft was the saphenous vein, while the cephalic vein was used when the saphenous vein was insufficient or unavailable. The cryopreserved allografts were obtained from Helsinki University Children's Hospital Homograft Bank. No synthetic grafts were used.

Pedicled muscle flaps with sartorius and gracilis were used for soft tissue coverage in three of the primary operations and microvascular LD in three. In two patients, no flap was initially used but the free flap reconstructions were performed on postoperative days 64 and 105 to solve wound healing problems. For the LD flaps, the entire muscle was used to fill the cavity in the defect and, if necessary for the wound closure, a skin island of 5–9 cm × 16–26 cm was incorporated.

The median operative time was 7.9 (4.3–9.1) h (Table 2). The median blood loss during surgery was 1565 (92–5000) ml, and 1 (0–14) units of red blood cells were transfused during the day of the surgery. Five of the patients were treated in the high dependency unit, most for 1 day. The median hospital stay was 10 (8–54) days. (Table 2).

**TABLE 2:** Perioperative details for patients with soft tissue sarcoma who underwent tumor removal and vascular reconstruction

ID	Duration of operation (h)	Blood volume loss during surgery (ml)	Units of red blood cells transfused (*n*)	Days in HDU	Days in hospital	Perioperative complications^a^ (Clavien‐Dindo class)
Patient 1	8.9	NA	NA	1	11	3b
Patient 2	8.1	860	2	1	24	3b
Patient 3	4.3	1565	1	0	9	3b
Patient 4	8.8	2035	2	1	10	None
Patient 5	5.3	1735	2	0	10	None
Patient 6	7.1	92	0	1	8	3a
Patient 7	7.7	1300	2	0	8	3b
Patient 8	9.1	5000	14	2	54	3b

Abbreviation: HDU, high dependency unit.

^a^ Within 30 days of surgery.

The thromboprophylaxis regime included perioperative intravenous heparin at a dose of 100 IU/kg and long‐term acetylsalicylic acid 100 mg once daily. Low molecular weight heparin was used postoperatively for up to 3 months. A double platelet inhibition with the addition of clopidogrel was introduced for 3 months after balloon angioplasty for graft stenosis.

The patients were recalled for clinical evaluation at the discretion of the senior author, with a special focus on the long‐term vessel patency and limb function. Duplex ultrasound was used to assess graft patency. The examination was done by an experienced vascular nurse specially trained in vascular graft surveillance.

The presence of limb edema was assessed at time of the last vascular follow‐up by a physiotherapist trained in the procedure with the truncated cone method with serial circumferential measurements at 4 cm intervals (Brorson et al., 2015). The oedema percentage was expressed as a percentage increase in limb volume in comparison with the healthy side. Only values below the knee were included to avoid confounding volume measurements arising from the tumor resection and reconstruction. The use of compression socks was recorded and judged as appropriate if at least two socks were in use alternatively and had been re‐measured every 6 months.

The Musculoskeletal Tumor Society (MSTS) 1993 score and the Toronto extremity salvage score (TESS), the two most commonly used functional outcomes scores in postoperative lower extremity sarcoma patients, were used for patient and clinician reported functional assessment (Davis et al., 1996; Enneking et al., 1993; Kask, Barner‐Rasmussen, Repo, Kjäldman, et al., 2019). The Finnish translations for these instruments have recently been validated (Kask et al., 2020; Kask, Barner‐Rasmussen, Repo, Blomqvist, & Tukiainen, 2019).

All the results are presented as median (range). IBM SPSS software was used for the analysis (IBM SPSS, 2017). The report was approved by the Helsinki University research ethics board and was conducted in accordance with the Helsinki Declaration.

## 3 RESULTS

The histological resection margin was wide, defined as a 2.5 cm or greater tumor free margin or excision with a natural tissue barrier, or marginal in seven patients and intralesional in one. In addition, surgical site contamination with the tumor occurred during the operation on the patient number 8.

**FIGURE 2:**
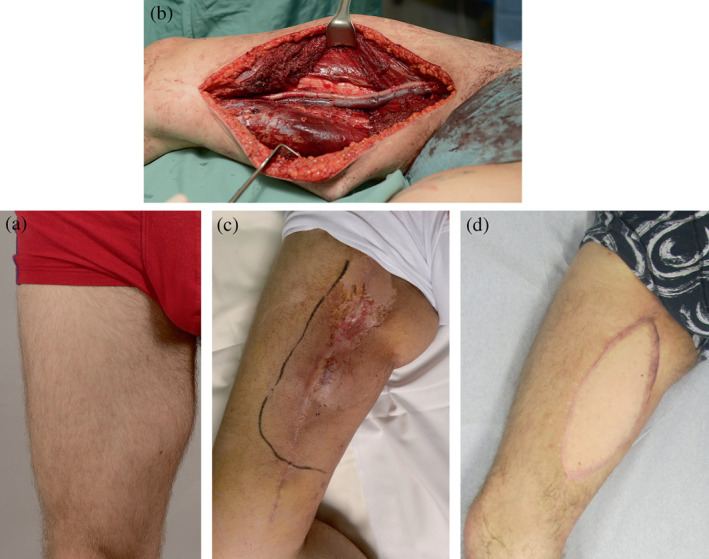
Patient 5. (a) Preoperative image; (b) perioperative image displaying reconstructed superficial femoral artery and vein that were initially covered with direct apposition of the muscles; (c) Seroma infection in the area following postoperative radiotherapy; (d) final result following revision and LD flap to fill the defect cavity

Five of the patients required a second operation within the 30‐day perioperative period and four later‐on for postoperative complications (Table 1). Three of the patients required more than one additional operation. An additional flap was done in three patients, two for complications related to lymphatic collections and one in the context of a surgical site infection. This included the patient number 5 who did not have a flap as part of the primary operation (Figure 2). The patient number 8 required repeat thrombectomies to the graft vein without long‐term patency, despite the use of venous compression devices and high dose low molecular weight heparin.

Assessment of graft patency with duplex ultrasound was available for seven patients with a follow up of 48 (2–76) months (Table 3). Five (71%) of the vein grafts had thrombosed. These included both of the autologous vein grafts and three of the five allografts.

**TABLE 3:** Graft patency and functional outcomes for patients with soft tissue sarcoma who underwent tumor removal and vascular reconstruction in Helsinki University Hospital from 2014 to 2020

ID	Time from surgery (months)	Graft patency	Lymphedema^a^ (%)	Compressive sock use/appropriate	MSTS (%)	TESS (%)	Gait
Patient 1	76	Artery patent, vein obstructed at 4 months	14	Yes/No	80	85	Normal
Patient 2	52	Artery patent, vein obstructed at 23 months	13	Yes/Yes	43	75	Unsteady, uses a crutch
Patient 3	48	Artery obstructed at 21 months, vein patent	2	No/No	70	89	Normal. Claudication at 40 m
Patient 4^b^							
Patient 5	48	Artery stenosed, vein obstructed at 6 months	144	Yes/Yes	70	90	Normal
Patient 6	33	Artery and vein patent	23	Yes/No	70	100	Normal
Patient 7	6	Artery patent, vein obstructed since last assessment	3	No/No	87	91	Normal
Patient 8^c^							

Abbreviations: MSTS, The Musculoskeletal Tumor Society 1993 score; TESS, The Toronto extremity salvage score.

^a^ Volume difference in comparison with the non‐operated side.

^b^ Lost to follow‐up.

^c^ Only discharged from hospital at time of writing. Venous graft obstructed on postoperative day 33, artery patent.

Five of the arterial grafts remained patent during follow‐up. The arterialized autologous vein graft of patient 3 had obstructed at 19 months, and, after an unsuccessful angioplasty, a new arterial bypass graft was done. The new graft was found obstructed with sufficient collateral circulation to maintain leg vitality at 23 months. At the 48‐month follow‐up, he reported a claudication distance of 40 m. The patient number 5 had several angioplasties for the arterial allograft starting at 7 months. At 48 months the graft was again found stenosed and an angioplasty was programmed.

The postoperative functional outcome was assessed clinically for six patients at 48 (6–76) months (Table 3). Three of the patients had oedema with more than 10% volume increase in comparison with the non‐operated leg, one despite appropriate use of compression garments. Reflecting an acceptable functional outcome, the median MSTS score was 70 (43–87)% and TESS 90 (75–100)%. Only patient 2 had an abnormal gait and used a crutch.

## 4 DISCUSSION

Soft tissue sarcomas surrounding or invading major blood vessels pose a surgical challenge as their radical resection often requires reconstruction of both the involved vessels and the soft tissue deficit. While oncovascular surgery aiming at limb salvage is emerging as an option in sarcoma surgery, the role of associated microvascular flap reconstructions is less well established (Adelani et al., 2007; Ghert et al., 2005; Miyamoto et al., 2017; Umezawa et al., 2013). In addition, few reports are available on the longs term functional outcomes achieved (Davis et al., 2017; Emori et al., 2012; Ghert et al., 2005; Schwarzbach et al., 2005). The proximal thigh, in particular, is a region where the resection of a comparatively small section of a muscle, the femoral nerve, or the lymphatics can result in a significant functional impairment of the leg distal to the operated area. Here we presented eight patients treated for soft tissue sarcomas in the proximal thigh or inguinal region in Helsinki University Hospital.

The limb was successfully salvaged in all eight patients with one local recurrence detected with a median follow‐up of 40 months. This is in line with the combined limb‐salvage rate of 94% reported in a recent review including 18 studies and 271 sarcoma patients with lower limb vascular reconstructions (Fujiki et al., 2020). The aim to achieve adequate disease control without above the knee amputation or hip disarticulation reflects the perceived functional advantage a reconstructed limb provides. Studies comparing the health‐related quality of life in sarcoma patients treated with limb‐sparing surgery and amputation have suggested that limb‐salvage surgery is associated with less functional and psychological handicap (Davis et al., 1999, 2017; Malek et al., 2012; Mei et al., 2014; Reijers et al., 2022). However, no overall quality of life benefit has been consistently observed (Mei et al., 2014). In our view, preserving the ability to perform tasks of daily living independently is an important goal even in patients with limited long‐term prognosis. Therefore, we do not consider high‐grade tumors or metastatic disease as an indication to favor more mutilating options over limb‐sparing surgery but emphasize case by case assessment.

The median MSTS score of the six patients assessed in our cohort was 70%, median 48 months after the operation. This is lower than previously reported in two small series for patients with lower limb sarcomas requiring oncovascular surgery (Davis et al., 2017; Emori et al., 2012). The median TESS score was 90%, slightly higher than reported elsewhere (Ghert et al., 2005). As the TESS is a self‐reported instrument measuring the ability to perform tasks of daily living, it appears our patients did not feel restricted by the physical limitations captured by the MSTS. Importantly, five of the six patients had re‐gained normal gait. For lower limb soft tissue sarcoma patients in general, a recent review reported postoperative MSTS and TESS scores of 83% and 86%, respectively (Kask, Barner‐Rasmussen, Repo, Kjäldman, et al., 2019). However, the small size of our cohort and the other reported series precludes any real inference. With functional outcomes scores generally not improving significantly after 1 year postoperatively, longer follow‐up is unlikely to have influenced our findings (Kask, Barner‐Rasmussen, Repo, Kjäldman, et al., 2019).

Autologous vein grafts were used whenever possible, saphenous vein when available and cephalic vein as the second option. Allografts were used only when no suitable vein was available for grafting. This reflects our preference to avoid the use of synthetic material at iliac vessels and distally, as well as in areas where the likelihood of delayed wound healing or postoperative infection is perceived high. In addition, the long‐term patency of venous grafts for arterial bypass in the femoral region supersedes that for PTFE grafts (Klinkert et al., 2004). A similar preference for autologous grafts has been reported by other centers (Baxter et al., 2007; Davis et al., 2017; Miyamoto et al., 2017; Nishinari et al., 2015; Okamoto et al., 2018; Poultsides et al., 2015; Umezawa et al., 2013). However, some surgeons routinely use PTFE graft as the first choice (Emori et al., 2012; Ghert et al., 2005; Schwarzbach et al., 2005). The use of allografts, although common in reconstruction of retroperitoneal vessels, appears rare (Homsy et al., 2020; Poultsides et al., 2015).

Duplex ultrasound assessment of the vascular graft patency was done for seven patients at median follow‐up of 48 months. One of the patients had an obstructed arterial graft while another required repeat angioplasties. The obstruction rate was higher for the vein grafts with five of the vein grafts thrombosed during the follow‐up and only two of the grafts unobstructed at 24 months of follow‐up. The arterial graft patency rates we observed were similar to, but the venous graft rates worse than, those reported elsewhere for sarcoma patients (Adelani et al., 2007; Fujiki et al., 2020; Nishinari et al., 2015; Okamoto et al., 2018; Poultsides et al., 2015). The risk factors for graft occlusion have not, to our knowledge, been studied in the context of sarcoma reconstruction. With arterial bypass grafts for arteriosclerosis, the graft patency is dependent on the distal vasculature, not properties of the graft (Albäck et al., 1998).

Notably, all resected femoral veins were reconstructed in our patients. This reflects our approach of reconstructing the superficial femoral vein, even when the deep femoral vein is left intact by the sarcoma resection. While most studies suggest a benefit from venous reconstruction at least in cases where two major veins of the lower limb have been resected and no collateral venous circulation is detected, some challenge the need for the reconstruction of femoral veins altogether (Adelani et al., 2007; Fujiki et al., 2020; Tsukushi et al., 2008). In our view, it is conceivable that the role of the vein reconstructions in reducing limb edema is most pertinent in the early postoperative period, while the later formation of new collaterals may mitigate the importance of the grafts on venous return. Thus, although graft occlusion is more frequently observed in the reconstructed veins than in the arteries, it seems better tolerated in the long run.

Edema is one of the major complications of proximal thigh resections in lower limb sarcoma patients. With major lymph vessels coursing along the femoral vessels and superficial vein, resection of these vascular structures severs also the lymphatic pathways. Thus, the edema results from disruption to both the lymphatics and the venous circulation and can be further exacerbated by adjuvant radiotherapy. Methods proposed to enhance lymph flow recovery following tissue reconstruction have the placement of the flap in an orientation where pre‐mapped lymphatic channels align axially with the lymphatic flow present at the defect site prior to resection, either with or without a lymphaticovenous anastomosis (Pereira et al., 2021; Scaglioni et al., 2021, 2022; Yamamoto et al., 2018, 2021). No lymphatic mapping was done prior to flap reconstructions in our patients. At least mild edema was present in four of the six patients evaluated, only two of whom used compression socks according to instructions. The presence or extent of edema is inconsistently reported in the literature for lower limb sarcoma with vascular reconstructions. Some series observed some degree of edema in all patients while others report edema only as a transient postoperative issue, minimized by venous reconstruction (Baxter et al., 2007; Ghert et al., 2005; Nishinari et al., 2015; Umezawa et al., 2013).

The perioperative morbidity associated with the oncovascular sarcoma surgery described here was high and the employed multidisciplinary resources significant. The median duration of the operation was almost 8 h and the blood loss during surgery 1565 ml. While the perioperative mortality was zero, five of the patients required high dependency unit treatment postoperatively. Elsewhere, in‐hospital mortality of up to 12.5% has been reported (Okamoto et al., 2018).

Significant perioperative morbidity was observed with six of the patients having a Clavien‐Dindo class 3 complication within 30 days of surgery (Dindo et al., 2004). Three of the re‐operations were done for surgical site infection, two of these in the context of an infected seroma. While none of the flaps were lost during follow‐up, additional flap reconstructions to improve soft tissue coverage were done in three patients. Notably, only one patient required interventions for graft occlusion during the perioperative period.

Our early experiences and reports of prolonged lymphatic collection despite meticulous ligation of the lymphatic vessels during the surgery and the resulting delayed wound closure have shaped our practice to favor immediate flap reconstruction, with a pedicled or a microvascular flap (Davis et al., 2017; Ghert et al., 2005). In this series, the patients number 2 and 5 illustrated how a seroma formation around the vessel reconstructions led to a later requirement for a flap despite the soft tissue coverage of the grafts having been judged sufficient in the primary operation. In addition, no patients with soft tissue sarcoma resection and vascular reconstruction without the need for a soft tissue reconstruction were identified. These observations support the incorporation of a muscular flap in the primary operation to cover the vessel grafts and to fill the created cavity to prevent wound healing complications.

The use of flaps is our present practice unless the grafts can be covered directly with surrounding muscles. When no pedicled flap of sufficient volume is available in the thigh, or preoperative radiotherapy renders the local flap less reliable, musculocutaneous LD is our preferred choice. Although the LD could be harvested without a skin component, the additional volume created by the muscle flap would often render a direct closure of the wound edges too tight. Thus, we frequently include a skin island in the flap, harvested in an axial orientation on the anterior border of the muscle, where a skin island of at least 9 cm wide can easily be harvested without difficulty in primary closure of the wound. The skin island also enables easy clinical monitoring of the flap perfusion. The arterial inflow to the flap is frequently connected to the arterial graft while a native vein is preferred for the venous outflow to minimize the circulatory compromise an obstructed venous graft would inflict on the microvascular flap. The microvascular surgery involved is relatively simple as the vessel diameter is large and the anastomosis site is well‐exposed.

Our report is limited by the small number of patients and the short follow‐up for some of them. In addition, no postoperative functional outcomes assessment was available for two of the eight patients, further limiting the population. The absence of a control group of either patients treated with amputation or those that did not require vascular reconstructions as part of their sarcoma removal precludes any comparative analysis.

## 5 CONCLUSIONS

In conclusion, oncovascular surgery combined with soft tissue reconstruction through local or microvascular flaps enables limb‐sparing surgery in patients with soft tissue sarcoma necessitating resection of major blood vessels in the inguinal region or the proximal thigh. Despite the high early morbidity, the long‐term functional outcomes are generally good.

## FUNDING INFORMATION

This work was supported by funding from Helsinki University Musculoskeletal and Plastic Surgery Research Centre.

## Data Availability

The data that support the findings of this study are available from the corresponding author upon reasonable request.
